# Clinical Utility of the Addition of Molecular Genetic Testing to Newborn Screening for Hemoglobinopathies for Confirmation of Alpha-Thalassemia Trait

**DOI:** 10.3390/ijns11010012

**Published:** 2025-02-07

**Authors:** Lisa M. Shook, Deidra Haygood, Charles T. Quinn

**Affiliations:** 1 Division of Hematology, Cincinnati Children’s Hospital Medical Center, Cincinnati, OH 45229, USA; deidra.haygood@cchmc.org (D.H.); charles.quinn@cchmc.org (C.T.Q.); 2 Department of Pediatrics, University of Cincinnati College of Medicine, Cincinnati, OH 45229, USA; 3 Erythrocyte Diagnostic Laboratory, Cincinnati Children’s Hospital Medical Center, Cincinnati, OH 45229, USA

**Keywords:** hemoglobinopathies, alpha-thalassemia, genotyping

## Abstract

Hemoglobinopathies are commonly detected by newborn screening (NBS). One of the most difficult to accurately diagnose is alpha-thalassemia, which is indicated by the presence of hemoglobin (Hb) Barts on NBS. This mixed methods study incorporated (1) an implementation and quality improvement project to demonstrate the clinical utility of genetic testing added to standard procedures for likely alpha-thalassemia trait and (2) a qualitative study to determine the related educational needs of primary care providers (PCPs). During a two-year period, we attempted to perform alpha-globin genetic testing for all newborns with an abnormal NBS result (an “FA + Barts” pattern). We conducted semi-structured interviews with seven PCPs for thematic abstraction. In sixty neonates with presumed Hb Barts on initial NBS who had genetic testing, three (5%) did not have alpha-thalassemia. The remaining 57 (95%) had an alpha-thalassemia trait genotype. Non-deletion alpha-thalassemia occurred in 5%. Eight (13%) had genotypes that substantially altered genetic counseling for the individual and family members. Race and ethnicity were poor surrogates for genotype. PCPs expressed a willingness to participate in NBS follow up but had little specific knowledge about alpha-thalassemia. The addition of genetic testing for likely alpha-thalassemia trait to NBS had very high clinical utility, supporting its use in standard clinical care. Whenever possible, education and genetic counseling should not be provided based on the detection of possible Hb Barts alone without subsequent specific genetic verification. Educational and outreach programs for both PCPs and families about the importance of testing and trait counseling are needed for ongoing improvement.

## 1. Introduction

Hemoglobinopathies are a heterogeneous group of red blood cell disorders that are commonly identified by newborn screening (NBS) [1,2,3] Alpha-thalassemia is prevalent worldwide, especially in African, Asian, and Middle Eastern populations, but also in the United States, which is predominantly a nation of immigrants. Alpha-thalassemia is caused by deletions, sequence variants, or both, of one or more of the four paired alpha-globin genes (αα/αα). Given its genetic complexity, it is one of the most difficult hemoglobinopathies to accurately diagnose, especially at birth. Most NBS programs worldwide include the detection of sickle cell disease, a different hemoglobinopathy. In contrast, depending on geographic region, severe forms of alpha-thalassemia may not be a formal target disease (e.g., a secondary finding or not reported) [4,5]. Despite this, secondary and incidental results, like alpha-thalassemia trait status, have clinical significance.

In alpha-thalassemia, hemoglobin Barts (Hb Barts) is present at birth but disappears after several months of age. The amount of Hb Barts detected, which can be quantified on screening (although not necessarily reported to clinicians) and confirmatory testing, can differentiate between a clinically significant condition, requiring ongoing monitoring and treatment (e.g., Hb H disease [−−/α−]) and the alpha-thalassemia silent carrier or trait states. A small amount of Hb Barts at birth (e.g., 2–5%) is consistent with trait but does not distinguish among trait genotypes. Although asymptomatic, these carrier or trait states require accurate education and genetic counseling for parents and families.

A deletion of one of the four alpha-globin genes is called the alpha-thalassemia silent carrier state (αα/−α). A deletion of two of the four alpha-globin genes is called alpha-thalassemia trait, which occurs in two classical forms that are clinically indistinguishable, *cis* deletions (αα/−−) or *trans* deletions (−α/−α), and can only be differentiated by genetic testing. The risk of Hb Barts hydrops fetalis syndrome (−−/−−) in the offspring of parents who both have thalassemia trait is either 0% or 25%, depending on their trait genotypes. This is a critical distinction. Classical Hb H disease is caused by a deletion of three of the four alpha-globin genes (−−/α−), with *cis* deletions on one allele.

While *cis* deletions are most common in people of Southeast Asian ancestry, they occur in other populations as well. Moreover, “race” (which is a sociopolitical construct), skin color, and geographic ancestry are at best crude surrogates for genotype, especially in the United States, where newborns increasingly have multiple, even unknown, geographic ancestries. There are also numerous non-deletion alpha-thalassemia determinants (e.g., mutations of the promoter region, stop codon, polyadenylation signal, and others) that add additional complexity to the classical deletional genotypes outlined above.

NBS laboratory methods and follow-up protocols differ by country, but the overall process includes confirmatory testing for abnormal initial results in a manner that is linked to education and genetic counseling and, if necessary, treatment. Given that the accuracy of genetic counseling for alpha-thalassemia trait depends entirely on the number and allelic configuration of alpha-globin gene mutations, genetic testing is clinically necessary. We have previously shown that the addition of genetic testing to NBS for sickle cell disease has high clinical utility [6,7]. Therefore, we performed an implementation study of alpha-globin gene locus analysis (best clinical practice) for all newborns with an abnormal NBS result indicating likely alpha-thalassemia. We also determined the educational needs of primary care providers in this process.

## 2. Materials and Methods

### 2.1. Setting and Study Design

In Ohio, the initial newborn screening test is performed on dried blood spots at the Ohio Department of Health (ODH) central laboratory using a combination of isoelectric focusing (IEF) and high-performance liquid chromatography (HPLC). HPLC (Bio-Rad Variant^TM^ nbs Newborn Screening System, Hercules, CA, USA) is performed first, and any abnormal finding (any Hb pattern other than “FA”) is further investigated by IEF (GE Multiphor™ II Electrophoresis Unit, Piscataway, NJ, USA).

Any abnormal hemoglobinopathy result is then required to be confirmed using a new blood specimen obtained by venipuncture at (or coordinated by) an ODH Regional Sickle Cell Services Program (RSCP). The RSCP for the ODH public health region 1, comprising eight counties in southwestern Ohio, is the Cincinnati Comprehensive Sickle Cell Center at Cincinnati Children’s Hospital Medical Center (CCHMC). For this 2-year study, any newborn in whom Hb Barts was detected in isolation (an “FA + Possible Barts” pattern) on this initial screen was included (study period April 2022 and April 2024). The amount of Hb Barts is not reported by the ODH on the first NBS.

At the time of the required venipuncture for confirmatory Hb electrophoresis, limited genetic testing was performed using the same blood specimen. Copy number variation analysis of the alpha-globin gene cluster was performed by multiplex ligation-dependent probe amplification SALSA^®^ MLPA^®^ Probemix P140-C1 HBA, MRC, Amsterdam, The Netherlands. Sequence analysis of the alpha-globin genes (*HBA1*, *HBA2*) was performed by Sanger sequencing, including all exons, intron-exon boundaries, and 5′- and 3′-untranslated regions. Hb analysis at the time of genetic testing included both capillary zone electrophoresis (Capillarys 2 Flex Piercing^®^, Sebia, Lisses, France) and IEF (GE Multiphor™ II Electrophoresis Unit, Piscataway, NJ, USA) for all “FA + Possible Barts” patterns.

Race and ethnicity were self-reported values in our electronic health record. Parents of newborns could choose a single descriptor (e.g., Asian, Black/African American, White), multiple descriptors, or not provide any descriptors. In addition to these descriptors, parents could report ethnicity as Hispanic or not Hispanic. Education and counseling of families were genotype specific. Newborns who had Hb Barts detected in combination with another hemoglobinopathy, such as sickle cell disease or sickle cell trait, were not studied.

For the qualitative study, semi-structured interviews were conducted by a trained moderator [8] with primary care providers (PCPs) in southwestern Ohio. Study participants were sourced via convenience sampling of any PCP who has made referrals for abnormal hemoglobinopathy results to the RSCP. Study participants were recruited via an invitation study email and flyer and a mailing to providers listed in the RSCP database. Interviews were conducted online utilizing the Zoom© web conference platform. Interviews were recorded and transcribed, coded by trained coders, and thematic analysis was used to identify themes [8,9]. Thematic saturation was reached when no new themes among the providers were elicited. There was consensus with feedback received among each individual semi-structured interview. A directed content analysis approach was then used to code qualitative data into themes [10]. Two trained study team members independently coded all the transcripts, and a third trained study team member was a reliability coder.

### 2.2. Ethical Considerations

The quality improvement project was exempt from formal review by the Institutional Review Board (IRB) at CCHMC, and the requirement for written informed consent was waived. The qualitative study was approved by the CCHMC IRB. Participants in the semi-structured interviews gave electronic informed consent, had the right to withdraw from the study at any time, and received modest remuneration for their time.

### 2.3. Statistical Methods

No a priori sample size was calculated. All consecutive patients with an “FA + Possible Barts” pattern on NBS were included in this 2-year sample. Summary statistics were generated without formal statistical hypothesis testing.

## 3. Results

### 3.1. Implementation and Quality Improvement

**Figure 1:**
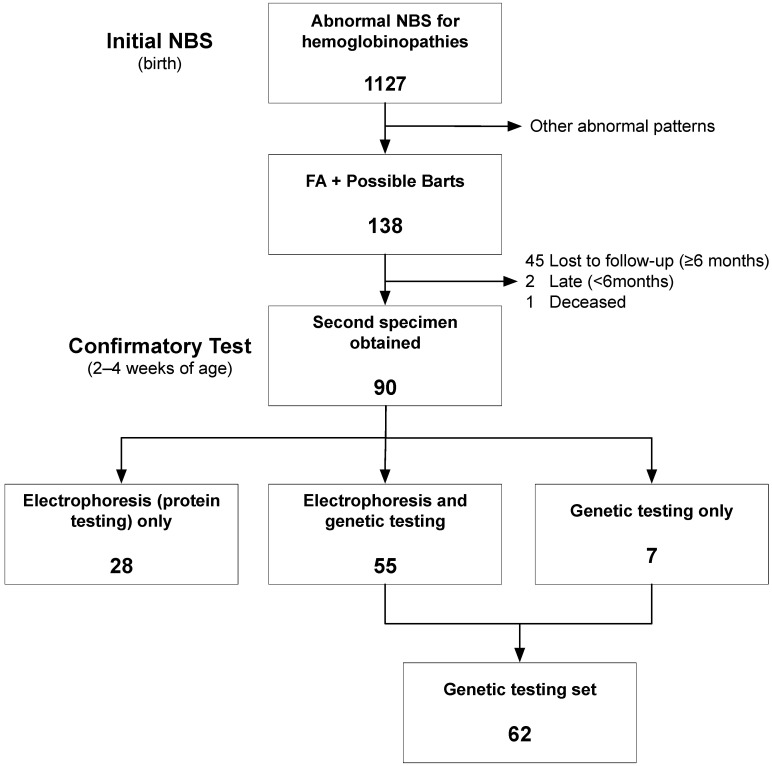
Newborns identified with possible hemoglobin (Hb) Barts on initial newborn screening. Between March 2022 and March 2024, there were 1127 abnormal initial newborn screening (NBS) tests for hemoglobinopathies. Of these, 138 had possible Hb Barts on the initial screen. The formation of the genetic testing set is shown.

During the study period, 138 newborns had an “FA + Possible Barts” pattern on the initial NBS specimen (Figure 1). Despite contacting families and arranging for a second screen to verify and quantify Hb Barts to exclude Hb H disease and confirm alpha-thalassemia trait, 45 (32.6%) newborns were lost to follow up (≥6 months). Although this is similar to our rates for other likely Hb traits (e.g., sickle cell trait and similar secondary findings on NBS), it contrasts with our loss to follow up rate of <1% for likely Hb disease cases (e.g., all genotypes of sickle cell disease). This high rate of loss to follow up for Hb traits presents a key opportunity for future improvement work.

During the study period, iterative methods and failure modes and effects analysis were used to integrate genetic testing into the established process for confirmatory testing (second NBS). The orders for genetic testing were placed by this study team upon notification from the ODH of Barts cases. However, PCPs throughout the community were oftentimes notified first and could order testing independently and without genetic testing. We worked to increase community awareness of this study (educational events, flowcharts, and mailings) and created standardized notes in the EMR to notify PCPs of the process. This reduced missing orders for genetic testing during the study period. We also found that laboratory personnel sometimes only processed the order for Hb electrophoresis or genetic testing (rather than both). We worked with laboratory leadership to increase awareness and also changed the ordering process to ensure that the orders for both testing components had the same date and time. This reduced incomplete testing (only Hb electrophoresis or only genetic testing) over the course of the study period. Through this iterative improvement process, we arrived at 62 newborns (69% of the newborns not lost to follow up or still awaiting testing) who had genetic testing for analysis (Figure 1; Supplemental Table S1).

### 3.2. Results of Genetic Testing

In these 62 neonates, we identified 65 globin gene mutations (pathogenic variants or protein-altering variants producing an abnormal Hb phenotype); these comprised 11 unique mutations (Table 1). Note that the non-alpha-globin variant (Table 1) has not yet been identified genetically but is included in the mutation total. 

Three (5%) neonates with possible Hb Barts on the initial NBS had other globin variants and not alpha-thalassemia (Table 1). Therefore, education and genetic counseling should not be provided based on the detection of possible Hb Barts without subsequent specific verification. The remaining 59 neonates (95%) had a genotype associated with an alpha-thalassemia trait phenotype (Table 2).

The majority of these fifty-nine neonates (81%) had 2 gene deletion alpha-thalassemia in trans (−α/−α), while seven (12%) had 2 gene deletion in *cis* (αα/−−). Of those with cis mutations, five had the common −−^SEA^ or −−^FIL^ deletions, but two were heterozygous for the less common 20.5 kb deletion (−−^20.5^) that includes the *HBA2* gene and the 5′ portion of the *HBA1* gene. Two neonates had compound heterozygosity for a single alpha-globin deletion and a non-deletion alpha-thalassemia determinant (Hb Constant Spring or a splicing site variant [α^Hph^]). Finally, one had a novel duplication involving the HS−40 enhancer region of the alpha-globin gene cluster. There were no cases of Hb H disease, other non-transfusion-dependent forms of alpha-thalassemia (NTDT; thalassemia intermedia), or Barts hydrops fetalis.

In this relatively small overall sample (*N* = 62) of neonates born in the Midwestern United States, in which there is limited diversity of geographic ancestry (predominantly European and African American), there was heterogeneity of alpha-thalassemia genotypes. Eight (13%) had genotypes that would substantially alter genetic counseling for the individual (i.e., individuals with 2 gene deletion in *cis* or a functionally similar genotype). These included five with −−^SEA^ or −−–^FIL/THAI^, which occur most commonly in individuals of Asian ancestry. There were also two patients with a 20.5 kb deletion (−−^20.5^) and one with a locus regulatory region (the DNase1-hypersensitive site 40 kb upstream of the ζ-globin gene (*HBZ*); HS−40) duplication (affecting the output of both alpha-globin genes on the same chromosome). Three newborns (5%) had non-deletion alpha-thalassemia variants (α^CS^α, α^Hph^α, and αα^HS−40^).

Race and ethnicity were poor surrogates for genotype (Table 3), especially for genotypes that required different counseling (i.e., 2 gene deletion in *cis* or a functionally similar genotype). Four out of five with the −−^SEA^ or −−–^FIL/THAI^ deletions identified as Asian; one identified as Black/African American. There were three individuals who identified as White; two had the −−^20.5^ deletion and one had an HS−40 variant. Before the availability of genetic testing, we would have counseled individuals of Asian ancestry (as a proxy for *cis* deletions) differently about their trait status compared to those of other geographic ancestries, and once genetic testing became available (before this study sample), we would have strongly offer testing for those of Asian ancestry. This past practice was clearly problematic. Counseling those of Asian ancestry about likely *cis* deletions is not always correct (here, 33% [2/6] did not have *cis* deletions), and preferentially offering testing to those of Asian ancestry misses other clinically significant alpha-thalassemia genotypes in other groups (here, 50% [4/8] of neonates would have been missed).

### 3.3. Qualitative Findings

For the qualitative study, seven PCPs participated in semi-structured interviews (Supplemental Table S2). A key theme relating to PCPs and their knowledge of genetic testing was how a better understanding of the process and its importance is essential for improving support for families (Supplemental Table S3).

## 4. Discussion

The addition of genetic testing for likely alpha-thalassemia trait to newborn screening had very high clinical utility, supporting its use in standard clinical care. Genetic findings substantially altered trait counseling for 13% of neonates in this study. We found high genetic heterogeneity of alpha-thalassemia, including deletion and non-deletion variants of the alpha-globin genes as well as a locus regulatory region variant. As such, the combination of sequence analysis and copy number variation analysis (for deletions, duplications, and rearrangements) was necessary for accurate diagnosis and counseling of the alpha-thalassemia trait states (and thalassemia, in general). Indeed, 5% of newborns had non-gene-deletion alpha-thalassemia alleles (α^CS^α, α^Hph^α, and αα^HS−40^).

We also found that classifications based on race (which is a sociopolitical, non-biological construct) and ethnicity (as dichotomized by our medical system) were poor surrogates for genotype. Indeed, 2 gene deletion alpha-thalassemia in *cis* (or a functionally similar genotype) was identified in similar numbers of individuals whose families identified as Asian (*N* = 4) or White (*N* = 3) in our population. We assert that these categories (“race” and ethnicity) should not be used to inform genetic counseling or to determine the need for genetic testing. Perhaps some unbiased assessment of geographic ancestry, if possible, could be used to estimate pre-test probabilities of certain genotypes, but we did not study that here.

We also found a substantial need for ongoing improvement. Over half of the neonates with likely alpha-thalassemia trait identified by NBS did not have confirmatory testing and counseling despite multiple attempts at contacting each family by our team. It appears that the necessity for such follow up is perceived as low by both medical providers in our community and the families. While not as critical as confirmation and follow up of disease states, like sickle cell anemia, hemoglobinopathy trait counseling is needed to inform family planning and provide education about expected mild phenotypic findings (e.g., microcytosis in thalassemia trait) that can be misinterpreted as another condition, such as iron deficiency. In the case of alpha-thalassemia trait, genotype-specific counseling about the risk to future offspring is needed, both for the neonates themselves (in the distant future) and the neonates’ parents who may have additional children. Indeed, the risk of Hb Barts hydrops fetalis syndrome (−−/−−) may be 0% or 25% in the offspring of parents who both have thalassemia trait, depending on the parents’ specific trait genotypes.

In the State of Ohio, NBS follow up is often driven by referrals from PCPs to specialty programs. Qualitative interviews with PCPs demonstrated that they are often unfamiliar with the processes of NBS in general and have “little to no experience” with genetic testing for hemoglobinopathies and would appreciate related education (Supplemental Table S3). While it is appropriate for NBS specialty programs to arrange genetic testing, PCPs expressed a willingness to understand its importance because they are seen by families as the “medical home” and a source of trustworthy medical information. It is notable that one PCP assigned a lower value to NBS follow up than many other potential concurrent clinical issues. This sense of non-urgency could be conveyed to families, decreasing their own priorities about testing or follow up. Therefore, both PCPs and families need to be included in ongoing educational and improvement efforts.

This is a relatively small study (*N* = 60 with genetic testing), so the heterogeneity of alpha-thalassemia genotypes in our target population is likely greater than we identified here. Moreover, the use of unbiased genetic testing (e.g., next-generation sequencing) instead of our combination of methods may have revealed even greater genetic heterogeneity of alpha-thalassemia. Our population is predominantly White/European and Black/African American, so genetic testing in populations with greater ethnic and geographic diversity may have even greater clinical utility. Additional studies are also needed to demonstrate the generalizability (including availability of timely testing) and cost effectiveness (especially in low-resource settings) of our approach. Finally, the processes for testing and follow up of NBS results vary widely across and within different nations and jurisdictions, so the processes reported here may not readily apply to other sites [3,4,5].

It is important to note that the overall goal of NBS programs for hemoglobinopathies in the US and Europe is the detection of sickle cell disease and not alpha-thalassemia [4,5,11]. Moreover, the mission of NBS programs generally does not include the detection of trait or carrier states that are mainly benign. However, when trait states are identified, clinicians are presented with medical information that is actionable, at the least by necessitating education and counseling. Given that genotype-specific counseling is needed for alpha-thalassemia trait, genetic testing can be incorporated into standard clinical care in different ways: addition to local medical practices after the reporting of NBS results, inclusion into national NBS programs for hemoglobinopathies (although this would require substantial, if not currently inordinate, programmatic changes across many jurisdictions), or a hybrid approach as we have implemented here.

In summary, genetic testing for likely alpha-thalassemia trait in the context of NBS had very high clinical utility, supporting its use in standard clinical care by enabling genotype-specific counseling. Ongoing interventions to decrease barriers to testing are needed, including the development of targeted education programs for both PCPs and families about the importance of testing and counseling.

## Figures and Tables

**Table 1:** Number of variant globin alleles identified by newborn screening.

Globin Variant	Number of Variant Alleles ^a^	Comment
α-thalassemia variants		
−α^3.7^	51	1 gene deletion
−−^SEA^	4	2 gene deletion (*cis*)
−−^20.5^	2	2 gene deletion (*cis*)
−−^FIL/THAI b^	1	2 gene deletion (*cis*)
−α^4.2^	1	1 gene deletion
α^CS^	1	Hb Constant Spring, *HBA2*: c.427T>C
α^Hph^ (α^+^)	1	Non-deletion α-thalassemia, *HBA2*: c.95+2_95+6del
HS−40 duplication	1	Non-deletion α-thalassemia (HS−40 variant)
Non-thalassemic variants		
Hb I-Texas	1	α-globin structural variant (benign)
Hb Stanleyville-II	1	α-globin structural variant (benign)
Normal α-globin locus	1	Likely γ-globin (fetal) variant (not yet identified)

^a^ Some individuals had more than 1 variant, so the total (*N* = 65) is greater than the population size (*N* = 62). ^b^ The −−^FIL^ and −−^THAI^ deletions cannot be differentiated by the technique used in the laboratory, but these 2 gene deletion forms of alpha-thalassemia would be managed/counseled identically.

**Table 2:** Alpha-globin genotypes of individuals (*N* = 62) identified by newborn screening.

Alpha-Globin Genotype	Number of Individuals	Zygosity	Comment
α-thalassemia genotypes			
−α^3.7^/−α^3.7^	48	homozygous	2 gene deletion (*trans*)
αα/−−^SEA^	4	heterozygous	2 gene deletion (*cis*)
αα/−−^20.5^	2	heterozygous	2 gene deletion (*cis*)
αα/−−^FIL/THAI a^	1	heterozygous	2 gene deletion (*cis*)
−α^3.7^/−α^4.2^	1	compound heterozygous	2 gene deletion (*trans*)
−α^3.7^/α^CS^α	1	compound heterozygous	1 gene deletion/Hb Constant Spring
−α^3.7^/α^Hph^α	1	compound heterozygous	1 gene deletion/α^Hph^ (*trans*)
αα/(αα)^HS−40^	1	heterozygous	Non-deletional variant
Non-thalassemic genotypes			
αα/α^Stanleyville-II^α	1	heterozygote	Hb Stanleyville-II heterozygote
αα/α^I-Texas^α	1	heterozygote	Hb I-Texas heterozygote
Normal α-globin locus	1	homozygous (normal α-globin locus)	Likely γ-globin (fetal) variant (not yet identified)

^a^ The −−^FIL^ and −−^THAI^ deletions cannot be differentiated by the technique used in the laboratory, but these 2 gene deletion forms of alpha-thalassemia would be managed/counseled identically.

**Table 3:** Self-reported ethnic and ancestral groups of newborns by alpha-thalassemia genotype (*N* = 59). This table excludes individuals with non-alpha-thalassemia genotypes (*N* = 3).

Genotype	Asian	Black/African American	Multiple	White	Not Provided
−α^3.7^/−α^3.7^	1	40	3	3	1 ^a^
αα/−−^SEA^	4	0	0	0	0
αα/−−^20.5^	0	0	0	2	0
αα/−−^FIL/THAI b^	0	1	0	0	0
−α^3.7^/−α^4.2^	0	1	0	0	0
−α^3.7^/α^CS^α	1	0	0	0	0
−α^3.7^/α^Hph^α	0	1	0	0	0
αα/(αα)^HS−40^	0	0	0	1	0

^a^ One individual reported Hispanic ethnicity. None of the other individuals reported Hispanic ethnicity. ^b^ The −−^FIL^ and −−^THAI^ deletions cannot be differentiated by the technique used in the laboratory, but these 2 gene deletion forms of alpha-thalassemia would be managed/counseled identically.

## Data Availability

The raw data supporting the conclusions of this article will be made available by the authors on request.

## Supplementary Materials

The following supporting information can be downloaded at https://www.mdpi.com/article/10.3390/ijns11010012/s1, Table S1: Demographic characteristics of the entire genetic testing population (*N* = 62); Table S2: Demographics of primary care provider interviewees; Table S3: Qualitative study themes.
