# Supplementary material for: Clinical Utility of the Addition of Molecular Genetic Testing to Newborn Screening for Hemoglobinopathies for Confirmation of Alpha-Thalassemia Trait
Source: Int J Neonatal Screen. 2025 Feb 7;11(1):12. doi: 10.3390/ijns11010012 (PMC11843879; doi:10.3390/ijns11010012)
Supplement: Supplementary file 1 [file IJNS-11-00012-s001.zip › IJNS-3348347-supplementary.pdf]

Supplemental Information

Supplemental Table S1. Demographic characteristics of the entire genetic testing population (N=62).

Characteristic	Value
Months of Age at genetic testing, mean (S.D.)	4.8 (4.5)
Sex, N. male/ N. female	27/35
Race, N.	
Black/African American	43
Asian	7
Not available or not provided	1
Multiple	3
White	8
Ethnicity, N.	
Hispanic	1
Not Hispanic	61

Abbreviations: N, number; S.D. standard deviation.

Supplemental Table S2. Demographics of primary care provider interviewees.

Demographic	N	%
Age		
Under 40	2	29%
40 – 49	4	57%
50 – 59	0	0%
60+	1	14%
Sex		
Female	7	100%
Practice Type		
Group	7	100%
Practice Location		
Urban	5	71%
Suburban	2	29%
Years in Practice		
<5	0	0%
6 – 10	3	43%
11 – 15	3	43%
16 – 20	0	0%
21 – 25	0	0%
>25	1	14%
Provider Type		
General Pediatrics	6	86%
Combined Internal Medicine / Pediatrics (Med / Peds)	1	14%

Professional Credentials		
MD	5	71%
DO	1	14%
DNP	1	14%
Patients Diagnosed with SCT		
<5	2	29%
6 – 10	1	14%
>10	4	57%
Patients Diagnosed with SCD		
<5	5	71%
6 – 10	2	29%
>10	0	0%

Supplemental Table S3: Qualitative Study Themes. The participants that were interviewed were a convenience sampling of PCPs (N=7) who contacted the program with questions about the NBS follow-up process, or who refer a high number of newborns with an abnormal screening result.

Theme: Providers are the medical home and have the relationships with patient families therefore it is important that the PCPs understand the newborn screening process, including genotyping.	*We're the medical home. These families will come back. Sometimes, they may ask us questions or maybe miss certain visits. And so, then they come and see us and then we're kind of that bridge. And so, I think it would be a helpful safety net to know what that is, to be able to explain it and maybe explain the importance of reconnecting with hematology or following up on that stuff. I think it would give us a little bit more confidence to be able to support the family*
Theme: PCPs can be a crucial bridge between families and the Sickle Cell Newborn Screening follow-up programs.	*"I think very important if it's going to be reliable and better for the family in the end. I think that's huge. And again, I think the role of the primary care doc is to help them get to the right place at the right time. Also, access to potentially if there's newer treatments as well. The sooner you get everything done I feel like the better. So, helping get it done I think. So, I think if we knew the importance and how accurate it was then I think we could advocate hopefully better to the family to get it done sooner."*
Theme: PCPs can assist with obtaining confirmatory testing blood draws if there is an understanding of what needs to be ordered.	*"… I think we could definitely help ensure they get to hematology for that testing. If the testing is super straight forward like a simple lab draw, and we can do it to expedite it only because families can get to us potentially quicker and more easily and we have more openings, I think we could do it if it's simple…*

Theme: PCPs may not understand the importance of possible Barts or the value of genotyping.	*"It is not as important as other things. Let's say on a scale of 0 to 10, like 10 being the most important thing and 0 being not important at all, I would probably say it's like a 6."*
